# Up-regulation of SIRT1 induced by 17beta-estradiol promotes autophagy and inhibits apoptosis in osteoblasts

**DOI:** 10.18632/aging.203639

**Published:** 2021-10-28

**Authors:** Yu Wang, Runhong Mei, Shimin Hao, Peng Luo, Penghao Wang, Yaser Almatari, Lei Guo, Lan Guo

**Affiliations:** 1 Department of Orthopedics, The First Hospital of China Medical University, Shenyang 110001, Liaoning, China; 2 Department of Orthopaedics, The Fourth Affiliated Hospital, Medical College, Nanchang University, Nanchang 330006, Jiangxi, China

**Keywords:** SIRT1, autophagy, apoptosis, AMPK-mTOR, FOXO3a

## Abstract

Osteoporosis is a common systemic skeletal metabolism disorder resulting in bone fragility and increased fracture risk. Silent information regulator factor 2 homolog 1 (SIRT1) is crucial in the regulation of several biological processes, including bone metabolism, autophagy, apoptosis, and aging. This study aimed to assess whether the up-regulation of SIRT1 induced by 17beta-estradiol (17β-E2) could promote autophagy and inhibit apoptosis in osteoblasts via the AMPK-mTOR and FOXO3a pathways, respectively. The study found that 17β-E2 (10^-6^ M) administration induced the up-regulation of SIRT1 in osteoblasts. Up-regulation of SIRT1 induced by 17β-E2 increased the expression level of LC3, Beclin-1, Bcl-2, p-AMPK, FOXO3a but decreased caspase-3 and p-mTOR expression, and then promoted autophagy and inhibited apoptosis. More autophagosomes were observed under a transmission electron microscope (TEM) in 17β-E2 and SRT1720 (a selective SIRT1 activator) co-treated group. When Ex527 (a SIRT1-specific inhibitor) was pretreated, the reversed changes were observed. Taken together, our findings demonstrated that the up-regulation of SIRT1 induced by 17β-E2 could promote autophagy via the AMPK-mTOR pathway and inhibit apoptosis via the FOXO3a activation in osteoblasts, and SIRT1 might become a more significant target in osteoporosis treatment.

## INTRODUCTION

Osteoporosis is a systemic bone disease, which is characterized by decreased bone mass and deterioration of bone microstructure, resulting in increased bone fragility and enhanced the risk of fracture [1]. More than 200 million people suffer from osteoporosis, and the incidence rate of osteoporosis is increasing with the prolongation of life span [2]. Over 70% of those over age 80 are affected. Epidemiology studies have shown that osteoporosis is more common in females than in males [3–5]. In the developed world, 9% to 38% of females and 2% to 8% of males are affected [6]. The incidence and severity of postmenopausal osteoporosis are associated with the aging process in conjunction with decreasing sex hormones, especially 17beta-estradiol (hereafter 17β-E2) [6, 7]. Estrogen/17β-E2 replacement has been proved to promote the differentiation of pre-osteoblasts into osteoblasts and prolong the life span of osteoblasts by curbing apoptosis [8, 9], suggesting 17β-E2 has a beneficial effect against osteoporosis. As is known to all, 17β-E2 binds to the specific nuclear estrogen-related receptors (ERRs) ERalpha and ERbeta to exert their physiological functions on cells [10, 11]. Both ERalpha and ERbeta are expressed in bone tissue [12, 13] and bind to 17β-E2 to regulate a multitude of physiological processes [10]. However, ERalpha is indeed the central factor in the osteoprotective effect of 17β-E2 [14]. It is reported that ERalpha plays a significant role in the bone response to estrogen in both men and women, while ERbeta only exerts a small protective role in women, but not in men [14, 15]. Dupont et al. found that deficiency of ERalpha was associated with decreased bone turnover and increased trabecular bone volume in male and female mice [16].

SIRT1, a class III histone deacetylase, participates in life span extension and age-related cellular mechanisms, including autophagy, apoptosis, energy metabolism and anti-aging [17–21]. Previous studies have shown that SIRT1 can reduce bone loss and promote bone formation in mice, indicating that it exerts a protective role in osteoporosis [22, 23]. *In vivo* and *in vitro*, experiments previously showed that SIRT1 promotes resveratrol-treated osteoblasts autophagy to protect against osteoporosis via activating the PI3K-Akt-mTOR signaling pathway [24]. Yao et al. have reported that overexpression of SIRT1 protects against osteoporosis by inhibiting apoptosis of osteoblast through the FOXO1 and β-catenin signaling pathway [25]. These studies have suggested that SIRT1 exerts a crucial protective role in osteoblasts and protects against osteoporosis.

Autophagy is an important mechanism of cellular homeostasis, and compromised autophagy has been proved to be related to the development of osteoporosis [26]. Several studies have suggested that osteoblasts show decreased autophagy in osteoporosis, leading to rapid cell apoptosis and bone mass loss [27–30]. Previous study suggested that AMP-activated protein kinase (AMPK) protects osteogenesis from ovariectomy-induced osteoporosis through autophagy [31]. In addition, AMPK has been proved to promote autophagy by inhibiting the mammalian target of rapamycin (mTOR) signaling pathway [32]. mTOR has been regarded as a potent inhibitor of autophagy. Whether 17β-E2 can upregulate SIRT1 expression and then promote osteoblasts autophagy through AMPK-mTOR signaling pathway has not been fully studied.

Previous study demonstrated that overexpression of SIRT1 protects the cardiomyocytes from oxidative stress via forkhead box O (FOXO) dependent mechanisms [33]. FOXO3a, an important member of the FOXO transcription factor family, has a definite anti-apoptotic and anti-oxidant stress effect [34]. SIRT1 inhibits apoptosis and resists oxidative stress via regulating FOXO3a [35]. Sun et al. reported that overexpression of SIRT1 in mesenchymal stem cells inhibits apoptosis and protects against bone loss in mice via FOXO3a deacetylation [36]. Therefore, we can hypothesize that 17β-E2 may induce up-regulation of SIRT1 and then inhibit osteoblasts apoptosis, which may be associated with FOXO3a activation.

In the present study, we sought to understand whether the up-regulation of SIRT1 induced by 17β-E2 could promote autophagy via the AMPK-mTOR signaling pathway and inhibit apoptosis via the FOXO3a activation in osteoblasts, and SIRT1 might become a more significant target in osteoporosis treatment. To evaluate the effect of SIRT1 in autophagy and apoptosis, osteoblasts were pretreated with or without SIRT1activator and inhibitor, and then the changes of autophagy- and apoptosis-related proteins were detected. Furthermore, the target molecules involved in modulating autophagy and apoptosis, such as AMPK-mTOR and FOXO3a, were also investigated.

## RESULTS

### Up-regulation of SIRT1 expression in hFOB1.19 osteoblasts induced by 17β-E2

**Figure 1:**
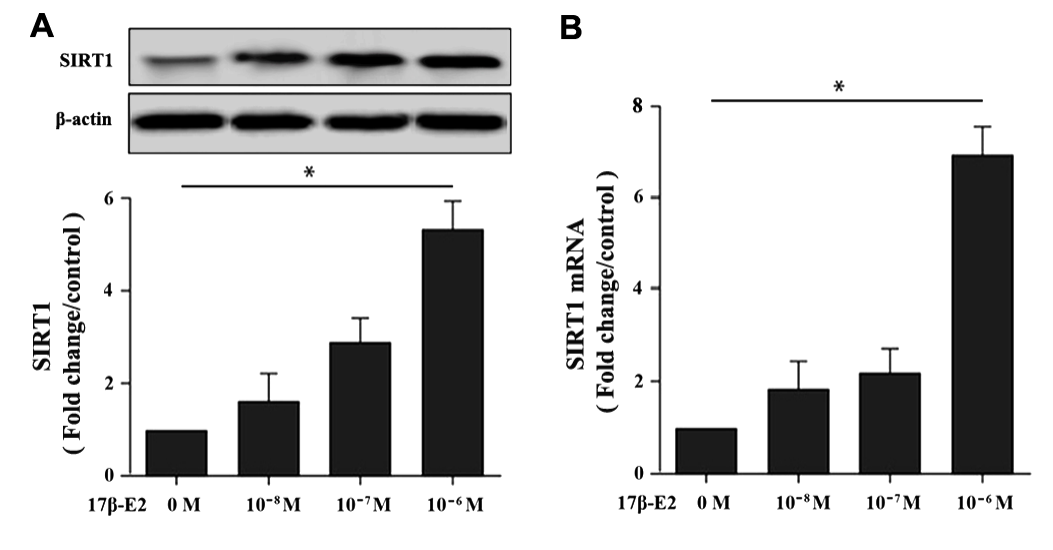
**Effect of 17β-E2 on the expression level of SIRT1 in hFOB1.19 osteoblasts.** (**A**) Western blotting showed the expression level of SIRT1 in osteoblasts treated with 17β-E2 (0, 10^-8^, 10^-7^, 10^-6^ M) for 24 h. Histogram showed the relative expression of SIRT1 normalized to β-actin protein, and the densitometry analysis of the blots were estimated by using Image J Software. (**B**) The expression of SIRT1 mRNA in osteoblasts treated with 17β-E2 (0, 10^-8^, 10^-7^, 10^-6^ M) for 24 h, and was assessed using RT-PCR. The data were normalized to GAPDH. Results were expressed as the means ± SD from 3 independent experiments. Statistically significance was evaluated using the Student’s t-test. * *P* < 0.05 vs. the control group was considered significant.

The hFOB1.19 cells have high homology with human osteoblasts, which is a good model system for studying the biological characteristics of normal osteoblasts *in vitro* [37]. To investigate whether up-regulation of SIRT1 in hFOB1.19 osteoblasts were induced by 17β-E2, cells were cultured with different dosages 17β-E2 (0, 10^-8^, 10^-7^ and 10^-6^ M) for 24 h. The expression of SIRT1 mRNA and protein were measured by Western blot (Figure 1A) and RT-PCR (Figure 1B). The results suggested that the expression of SIRT1 was upregulated remarkable after cells incubation with 17β-E2 (10^-6^ M) compared with the control group.

### 17β-E2 promoted autophagy in hFOB1.19 osteoblasts

**Figure 2:**
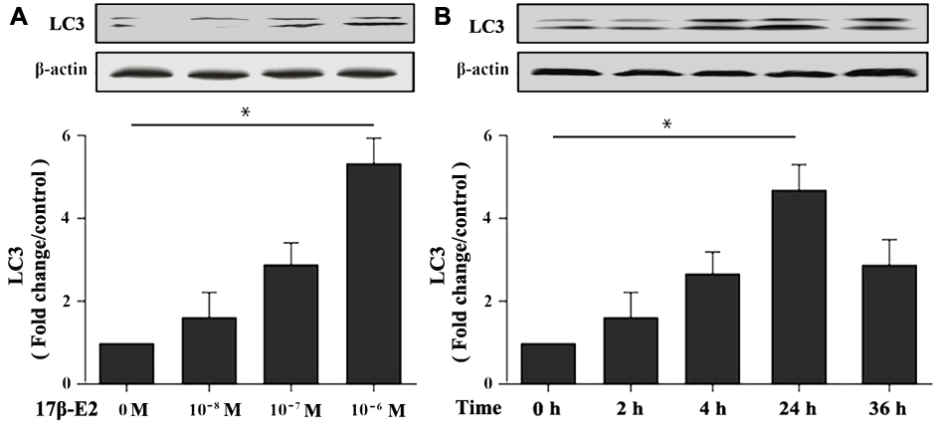
**Effect of 17β-E2 on the expression level of LC3 in hFOB1.19 osteoblasts.** (**A**) Western blotting showed the expression of LC3 protein in osteoblasts treated with 17β-E2 (0, 10^-8^, 10^-7^, 10^-6^ M) for 24 h. (**B**) Western blotting showed the expression of LC3 in osteoblasts treated with 10^-6^ M 17β-E2 for different time (0, 2, 4, 24, 36 h). The histograms showed the relative expression level of LC3 normalized to β-actin, and the densitometry analysis of the blots were estimated by using the Image J Software. The data were expressed as the means ± SD from 3 independent experiments. Statistically significance was evaluated using the Student’s t-test. * *P* < 0.05 vs. the control group was considered significant.

**Figure 3:**
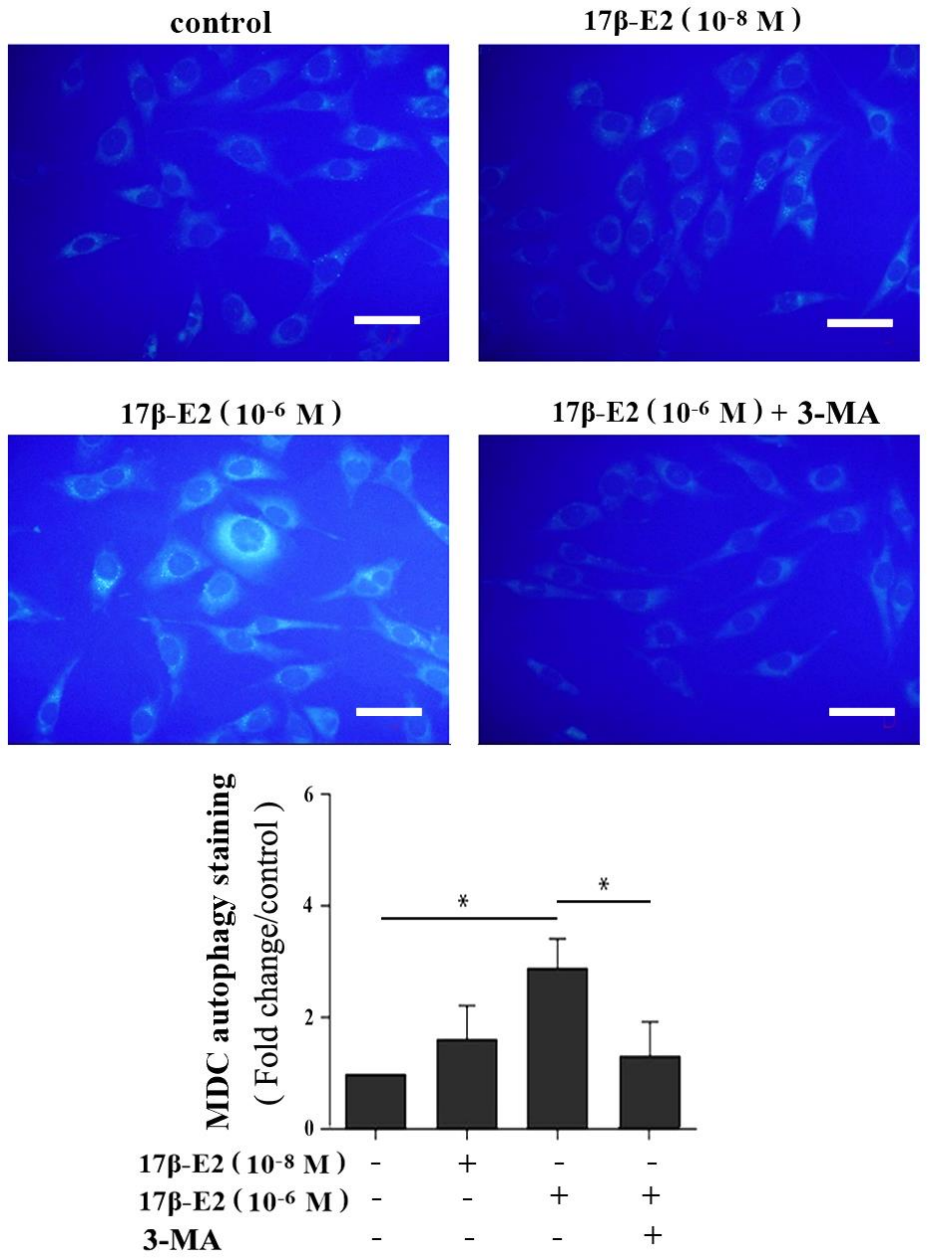
**Assessment of autophagy regulated by 17β-E2 in osteoblasts using monodansylcadaverine (MDC) staining.** The osteoblasts were pretreated with or without 3-MA for 2 h, and then cultured with 17β-E2 (0, 10^-8^, 10^-6^ M) for 24 h. Level of cell autophagy was evaluated by the intensity of fluorescent dots. The histogram showed that the relative intensity of fluorescent dots was significantly greater in 10^-6^ M 17β-E2 group than in the control group and 17β-E2 (10^-6^ M) + 3-MA group. Scale bar = 20 μm. The data were expressed as the means ± SD from 3 independent experiments. Statistically significance was evaluated using the Student’s t-test. * P < 0.05 vs. the control group was considered significant.

To investigate the effect of 17β-E2 on autophagy of hFOB1.19 osteoblasts, cells were cultured with different dosages of 17β-E2 (0, 10^-8^, 10^-7^ and 10^-6^ M) for 24 hours, and then the expression of autophagy marker microtubule-associated protein 1 light chain 3 (LC3) was measured by Western blot. The results revealed that the expression of LC3 in osteoblasts cultured with 10^-6^ M 17β-E2 was significantly increased than that in the control group (Figure 2A). Osteoblasts were treated with 10^-6^ M 17β-E2 for different time (0, 2, 4, 24, 32 h). Similarly, when cells were cultured with 10^-6^ M 17β-E2 for 24 h, the expression of LC3 was significantly increased compared with the control group (Figure 2B). Furthermore, osteoblasts were pretreated with or without 3-MA (an autophagy inhibitor) prior to incubation with 17β-E2 (10^-8^ and 10^-6^ M) for 24 h. It is reported that monodansylcadaverine (MDC) staining was considered to be a tracer of autophagosomes to detect autophagy [38, 39]. The change in the intensity of autophagy was detected by MDC staining. The MDC results suggested that the cells autophagy activity significantly increased in 17β-E2 (10^-6^ M) group, while it diminished in 17β-E2 + 3-MA group (Figure 3). Collectively, these results indicated that 17β-E2 could promote autophagy in hFOB1.19 osteoblasts.

### Up-regulation of SIRT1 induced by 17β-E2 promotes autophagy in osteoblasts

**Figure 4:**
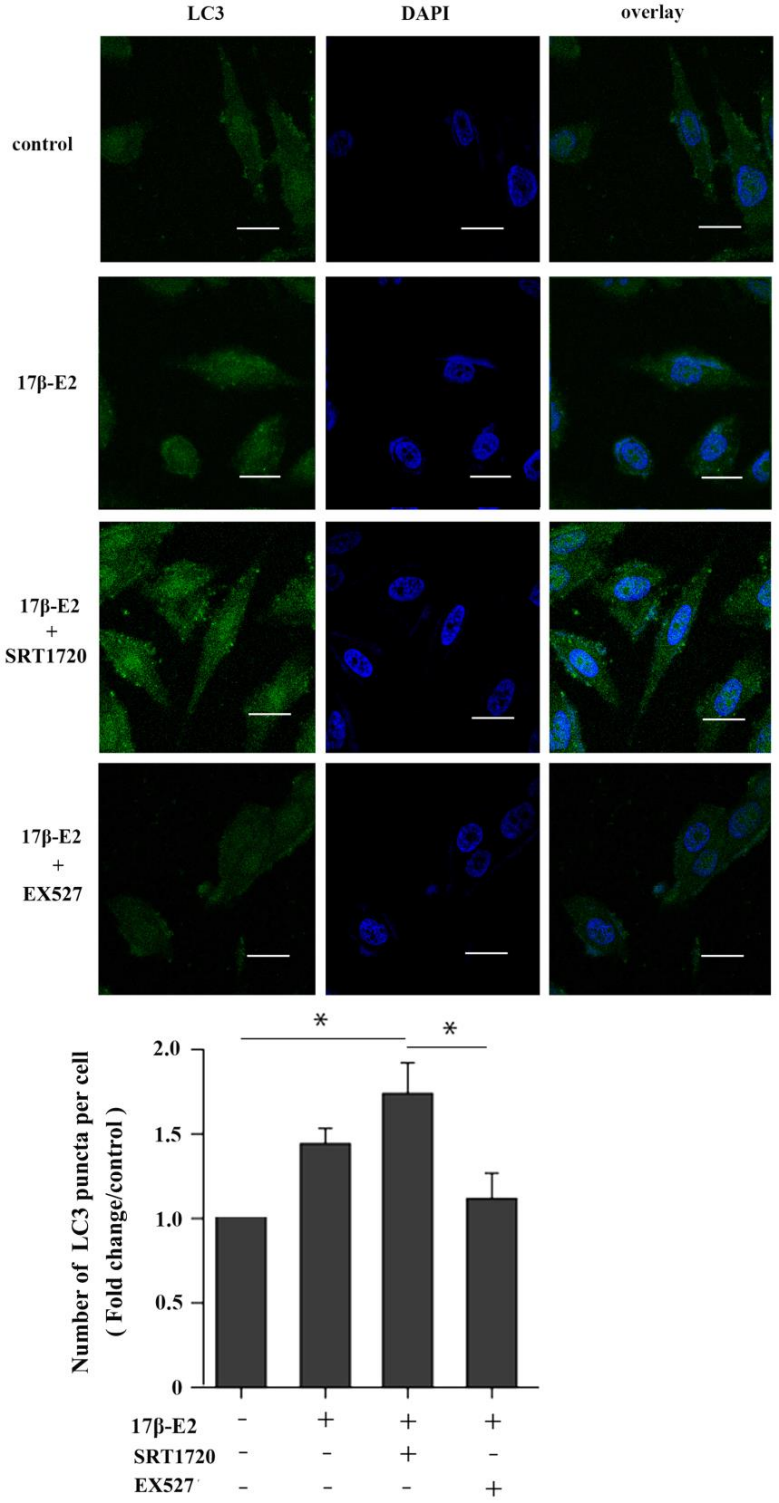
**Effect of SIRT1 up-regulation induced by17β-E2 on the expression of LC3 in hFOB1.19 osteoblasts.** SRT1720 or EX527 regulated the expression of LC3 in 17β-E2-treated osteoblasts, as determined using immunofluorescence staining. Nuclei were stained with DAPI (blue). Representative images and quantified analysis of immunofluorescence staining showing the LC3 in cells. White bar = 50 μm. The histogram showed the number of LC3 puncta in various groups as indicated. The data were expressed as the means ± SD from 3 independent experiments. Statistically significance was evaluated using the Student’s t-test. * *P* < 0.05 vs. the control group was considered significant.

**Figure 5:**
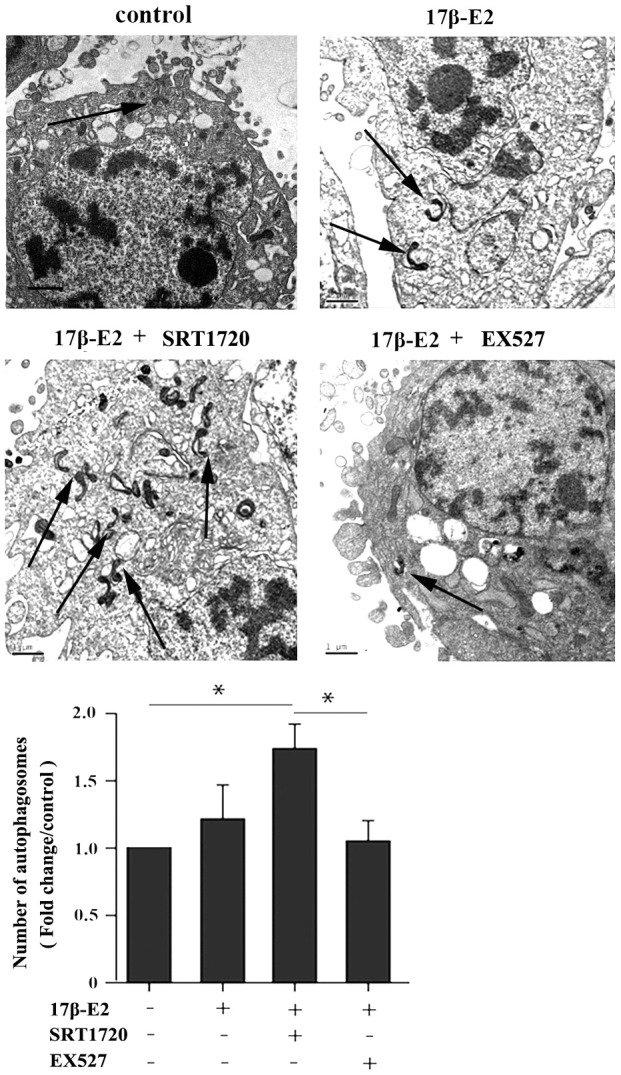
**Effect of SIRT1 up-regulation induced by 17β-E2 on cell autophagy in hFOB1.19 osteoblasts.** TEM images showed more double-membrane vacuoles (autophagosomes) in cells treated with 17β-E2 + SRT1720 compared with those in the control group and 17β-E2 + EX527 group. The histogram showed the number of autophagosomes in different groups as indicated. The autophagic vacuoles are indicated with black arrows. Black bar: 1 μm. * *P* < 0.05 vs. the control group was considered significant.

This study further explored whether up-regulation of SIRT1 induced by 17β-E2 could promote autophagy in hFOB1.19 osteoblasts. The cells were preincubated with or without SRT1720 (a SIRT1 activator) [40] and EX527 (a SIRT1-specific inhibitor) [41] for 2 h, and then they were incubated with 10^-6^ M 17β-E2 for 24 h. Immunofluorescence staining suggested that the expression of LC3 (marker of autophagy) in 17β-E2 + SRT1720 group was significantly increased than that in the control group (Figure 4). However, the increasing effect was abolished in 17β-E2 + EX527 group. TEM was considered to be an effective method to detect autophagy. TEM was served as a tool to observe the characteristics and quantify the number of autophagosomes. The results showed that the number of autophagosomes in 17β-E2 + SRT1720 group was significantly increased than that in control group while that in 17β-E2+EX527 group was significantly lower (*P* < 0.05), (Figure 5). To sum up, these results indicated that the 17β-E2 might induce SIRT1 up-regulation to promote autophagy in hFOB1.19 osteoblasts.

### Up-regulation of SIRT1 induced by 17β-E2 inhibits apoptosis in osteoblasts

**Figure 6:**
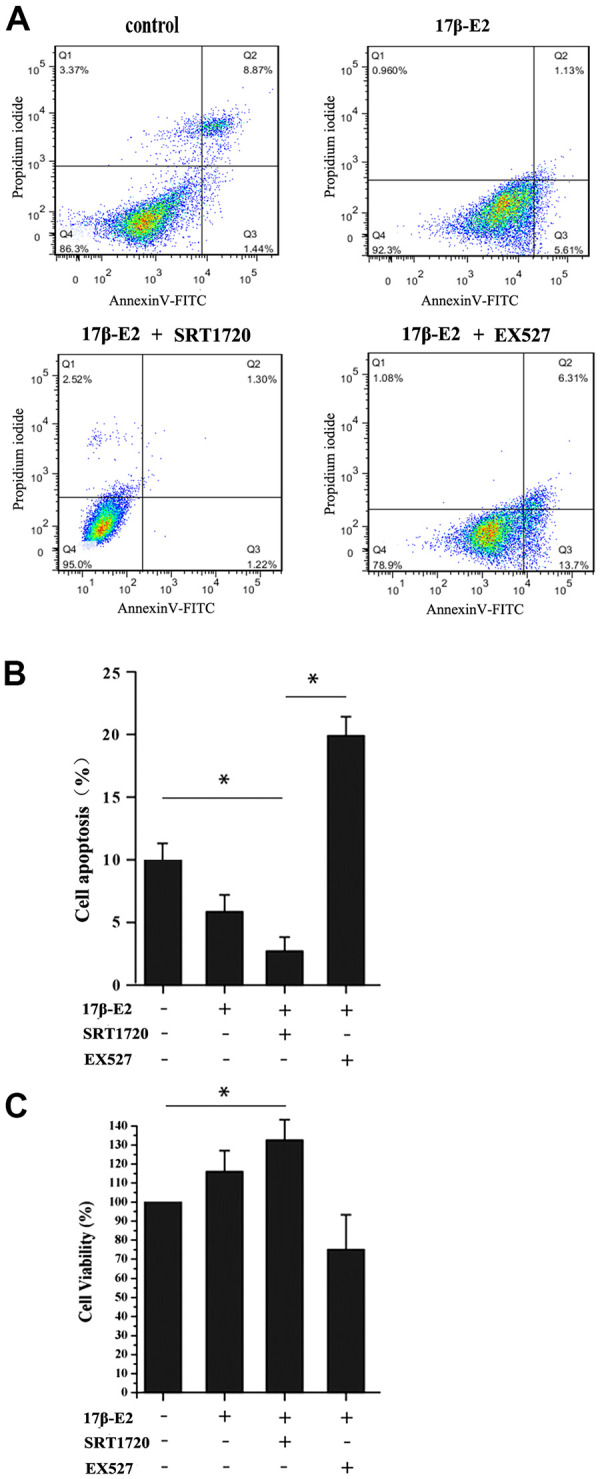
**Up-regulation of SIRT1 induced by 17β-E2 inhibited apoptosis in osteoblasts.** (**A**) Cell apoptosis rates (%) were measured by using flow cytometry (FCM) analysis of Annexin V-FITC/PI dual staining in osteoblasts pretreated with or without SRT1720 and EX527 for 2 h prior to incubation with 17β-E2 (10^-6^ M) for 24 h. Q1, dead cells; Q2, later apoptotic cells; Q3, early apoptotic cells; Q4, living cells. The apoptotic rate was determined as the percentage of Q2+Q3. (**B**) The histogram showed the percentage of apoptosis rate in different groups. The results suggested that the apoptosis rates (Q2+Q3) were significantly decreased in 17β-E2 + SRT1720 group compared with those in the control group and 17β-E2 + EX527 group. (**C**) MTT assay was performed to detect the osteoblasts viability, and the histogram showed the cell viability (%) in different groups. The data were expressed as the means ± SD from 3 independent experiments. * *P* < 0.05 vs. the control group was considered significant.

**Figure 7:**
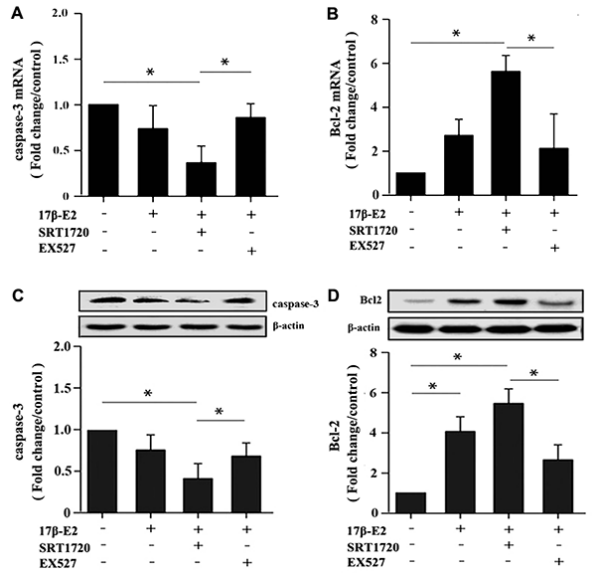
**Detection of caspase-3 and Bcl-2 expression in hFOB1.19 osteoblasts by real-time PCR and Western blot assay.** Cells were pretreated with or without SRT1720 and EX527 for 2 h, and then cultured with 17β-E2 (10^-6^ M) for 24 h. The expression of caspase-3 (**A**) and Bcl-2 (**B**) mRNA in osteoblasts were assessed by RT-PCR. The data were normalized to GAPDH. The expression level of caspase-3 (**C**) and Bcl-2 (**D**) proteins in osteoblasts were measured by Western blotting. The histograms showed the relative protein expression of caspase-3 (**C**) and Bcl-2 (**D**) normalized to β-actin protein, and the densitometry analysis of the blots were estimated by using the Image J Software. The data were expressed as the means ± SD from 3 independent experiments. Statistically significance was evaluated using the Student’s t-test. * *P <* 0.05 vs. the control group was considered significant.

hFOB1.19 osteoblasts were pretreated with or without 10 μM SRT1720 and 10 μM EX527 for 2 h, and then cultured with 10^-6^ M 17β-E2 for 24 h. Flow cytometry analysis using Annexin V-FITC/PI dual staining, quantitative RT-PCR and Western blot assay were applied to detect and quantify the level of apoptosis in hFOB1.19 cells. The apoptotic rate (Q2+Q3) decreased significantly in 17β-E2 + SRT1720 group, but increased in 17β-E2 + EX527 group (Figure 6A, 6B). The cell viability (%) in different groups was assessed by MTT assay (Figure 6C). The results suggested that the osteoblasts viability in 17β-E2 + SRT1720 group increased significantly compared with the control group, however this effect disappeared when exposure to a SIRT1-specific inhibitor EX527. The RT-PCR and Western blot results suggested that the expression of pro-apoptotic caspase-3 mRNA and protein decreased significantly, while the level of anti-apoptotic Bcl-2 increased in 17β-E2 + SRT1720 group compared with the control group (Figure 7A–7D). In short, the results suggested that up-regulation of SIRT1 induced by 17β-E2 inhibited apoptosis in hFOB1.19 osteoblasts.

### Up-regulation of SIRT1 induced by 17β-E2 promotes autophagy and inhibits apoptosis in osteoblasts

**Figure 8:**
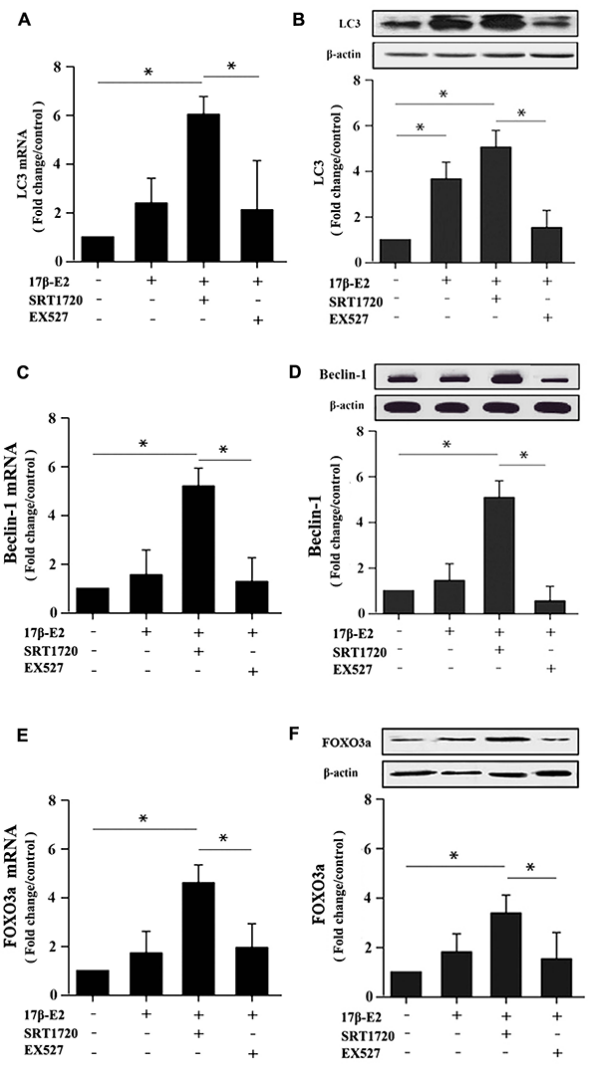
**Real-time PCR and Western blotting showing the expression of LC3, Beclin-1 and FOXO3a in hFOB1.19 osteoblasts.** Cells were pretreated with or without SRT1720 and EX527 for 2 h, and then cultured with 17β-E2 (10^-6^ M) for 24 h. RT-PCR and Western blot assay showed the expression of LC3 (**A, B**), Beclin-1 (**C, D**) and FOXO3a (**E, F**) mRNA and proteins in osteoblasts. The mRNA data were normalized to GAPDH. The histograms showed the relative proteins expression of LC3, Beclin-1 and FOXO3a normalized to β-actin, and the densitometry analysis of the blots were estimated by using the Image J Software. The data were expressed as the means ± SD from 3 independent experiments. Statistically significance was evaluated using the Student±s t-test. * *P* < 0.05 vs. the control group was considered significant.

**Figure 9:**
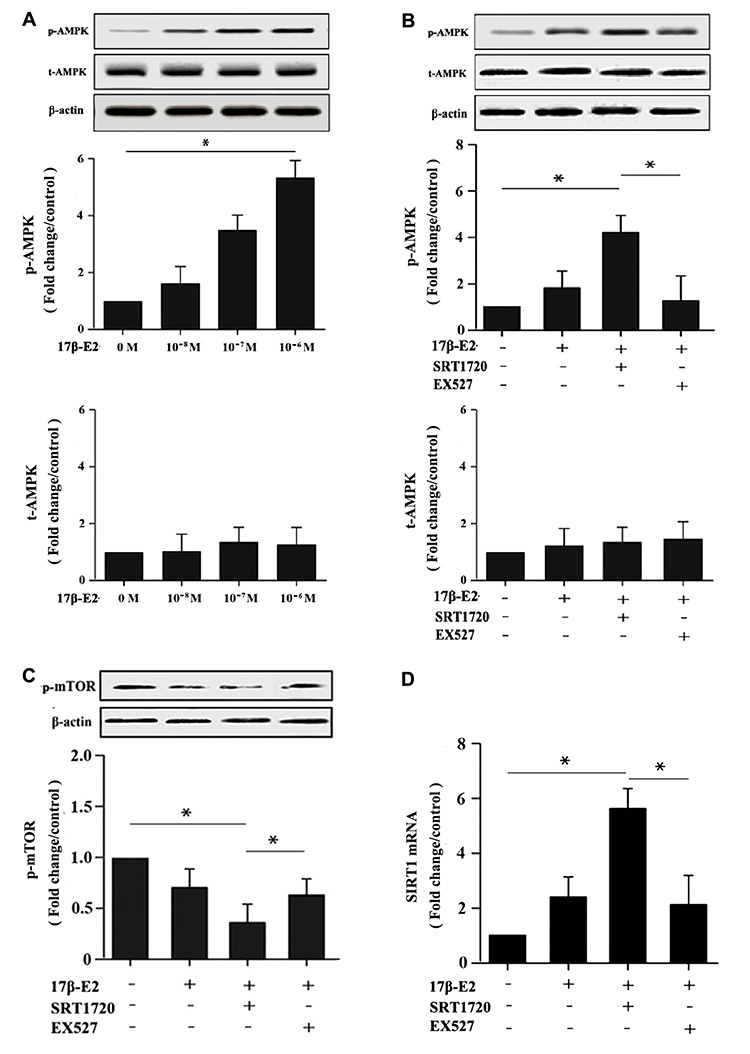
**Up-regulation of SIRT1 induced by 17β-E2 promoted autophagy in osteoblasts via AMPK-mTOR pathway.** Western blotting showed the expression of p-AMPK, t-AMPK, p-mTOR and β-actin in osteoblasts among the groups. (**A**) The expression level of AMPK phosphorylation in osteoblasts treated with 17β-E2 (0, 10^-8^, 10^-7^, 10^-6^ M) for 24 h. Cells were pretreated with or without SRT1720 and EX527 for 2 h, and then cultured with 17β-E2 (10^-6^ M) for 24 h. Western blotting showed the expression of p-AMPK (**B**), and p-mTOR (**C**) proteins in osteoblasts. The histograms showed the relative expression of p-AMPK and p-mTOR normalized to β-actin, and the densitometry analysis of the blots were estimated by using the Image J Software. The expression ofSIRT1 (**D**) mRNA in osteoblasts were measured by RT-PCR. The data were expressed as the means ± SD from 3 independent experiments. Statistically significance was evaluated using the Student’s t-test. * *P* < 0.05 vs. the control group was considered significant.

**Figure 10:**
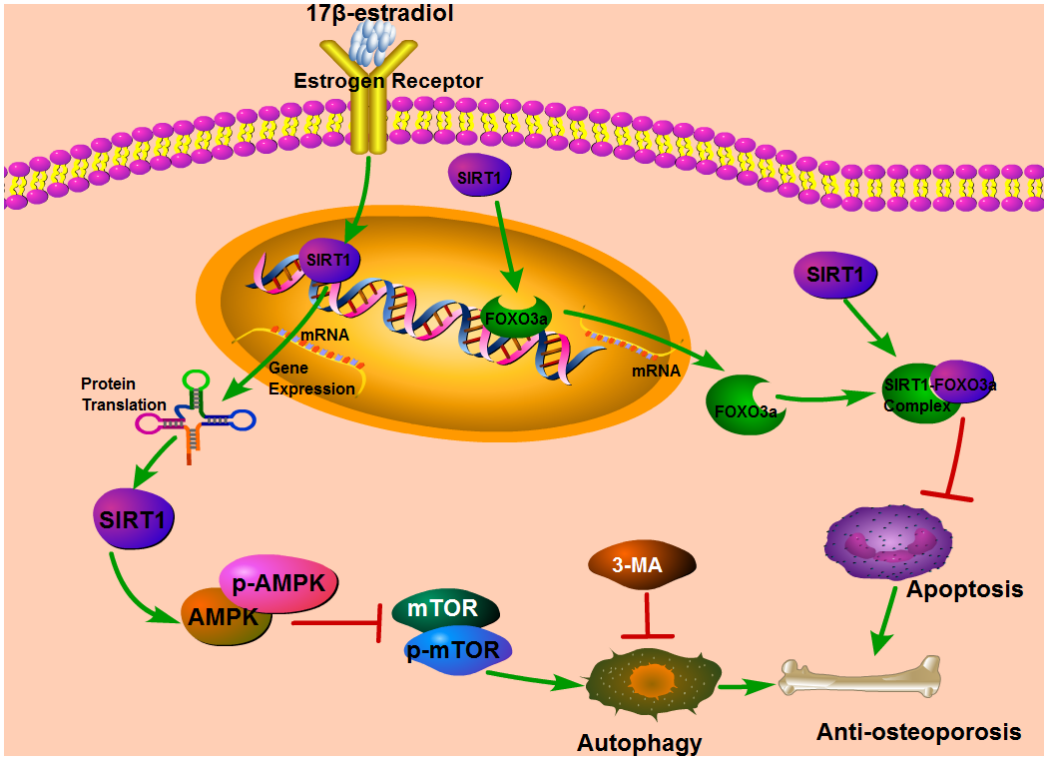
The diagram describes the underlying mechanism that the up-regulation of SIRT1 induced by 17β-E2 could promote autophagy via the AMPK-mTOR pathway and inhibit apoptosis by the FOXO3a activation.

Our results showed that up-regulation of SIRT1 induced by 17β-E2 promoted autophagy and inhibited apoptosis in osteoblasts. Here, in order to expound the molecular mechanisms of these processes, we measured the expression of the AMPK-mTOR and FOXO3a. Cells were precultured with or without SRT1720 and Ex527 prior to exposure to 10^-6^ M 17β-E2 for 24 h. The mRNA and protein expression levels of LC3, Beclin-1, caspase-3, Bcl-2, p-AMPK, FOXO3a, p-mTOR were measured by RT-PCR and Western blot assay. Compared with the control group, the expression level of LC3 (Figure 8A, 8B), Beclin-1 (Figure 8C, 8D) and p-AMPK (Figure 9B) increased remarkably in 17β-E2 + SRT1720 group, however a robust decrease in p-mTOR (Figure 9C). Notably, this action could be abolished by the SIRT1-specific inhibitor Ex527 and hardly be turned backward by 17β-E2. The expression of t-AMPK had no significant change in different groups (Figure 9A, 9B). The expression of caspase-3 in 17β-E2 + SRT1720 group was significantly decreased, yet a significantly increased in Bcl-2 compared with the control group (Figure 7A–7D). EX527 treatment resulted in the opposite effects. In addition, compared with the control group, the FOXO3a expression was significantly activated in 17β-E2 + SRT1720 group, while the expression of FOXO3a reduced in 17β-E2 + EX527 group (Figure 8E, 8F). Collectively, the results suggested that up-regulation of SIRT1 induced by 17β-E2 to promote autophagy and inhibit apoptosis may be associated with AMPK-mTOR and FOXO3a pathways, respectively (Figure 10).

## DISCUSSION

Postmenopausal osteoporosis (PMO) is the most common type of osteoporosis caused by estrogen deficiency in clinic [42]. The lack of estrogen has detrimental effects on all types of bone cells, especially osteoblasts, resulting in an increase in the level of bone turnover [42]. Many studies have shown that ERalpha plays a central role in the osteoprotective effects of 17β-E2 [14, 43, 44]. Vinel et al. indicated that ERalpha is necessary for the protective effects of 17β-E2 in the alveolar, trabecular and cortical bone of the mandible, while ERbeta is not indispensable [45]. Khan et al. reported that 17β-E2 positively regulates SIRT1 through ERalpha, thereby reducing memory loss in adult mice mediated by oxidative stress, neurodegeneration and neuroinflammation [46]. This study suggested that exposure to 17β-E2 induced up-regulation of SIRT1 in hFOB1.19 osteoblasts, leading to a promotion in cell autophagy and inhibition in cell apoptosis. Furthermore, our data showed that the effect of SIRT1 up-regulation positively regulated AMPK and FOXO3a, but inhibited mTOR. It is reported that the AMPK-mTOR signaling pathway regulates autophagy [47] and FOXO3a participates in apoptosis [35]. Autophagy and apoptosis play significant roles in osteoporosis [27]. Therefore, we could hypothesize that the up-regulation of SIRT1 induced by 17β-E2 protected against osteoporosis, at least in part, by promoting autophagy via AMPK-mTOR pathway and by inhibiting apoptosis via FOXO3a activation (Figure 10).

SIRT1, the mammalian homologue 1 of silent information regulator 2, plays important roles in the regulation of multiple biological processes, including bone metabolism, autophagy, apoptosis, and aging [20, 25, 48, 49]. Accumulating evidence has demonstrated that SIRT1 has a crucial effect in anti-osteoporosis through a complex signaling network, including autophagy [20] and apoptosis [50]. Zainabadi et al. reported that SIRT1 is not only a positive regulating factor of bone mass *in vivo*, but also a therapeutic target for osteoporosis [22]. In our experiments, the data suggested that 17β-E2 induced up-regulation of SIRT1 in hFOB1.19 osteoblasts. We also observed that up-regulation of SIRT1 participated in the promotion of osteoblasts autophagy. Gu et al. reported that SIRT1 protects osteoblasts from fluoride toxicity through the induction of autophagy [51]. Several studies also have reported that 17β-E2 can upregulate SIRT1 expression and play a beneficial role in osteoblasts [52, 53], which is accordance with our results. AMPK was regarded as a significant pathway of regulating cellular energy and metabolism homeostasis [54, 55]. It is well established that AMPK activation inhibits mTOR, thus promoting autophagy [32, 56]. The present study demonstrated that up-regulation of SIRT1 might activate AMPK and inhibit mTOR in osteoblasts under 17β-E2-induced conditions, and that the autophagy regulation effect of AMPK-mTOR signaling was attenuated when EX527 was added. Wang et al. demonstrated that tetramethylpyrazine could promote autophagy and protect bone marrow mesenchymal stem cells (BMSCs) from exposure to excessive glucocorticoids by the AMPK-mTOR signaling pathway [29]. Taken together, we hypothesized that up-regulation of SIRT1 induced by 17β-E2 could promote osteoblasts autophagy, which might be related to the AMPK-mTOR pathway.

Multiple lines of evidence have demonstrated that osteoblast apoptosis played an unfavorable role in the occurrence and progression of osteoporosis [42, 57, 58]. As is known to all, the anti-apoptosis ability of SIRT1 is self-evident [25, 51, 59]. Several studies showed that moderate SIRT1 overexpression could be effective in preventing drug induced apoptosis and oxidative stress [33, 60, 61]. In this study, the results suggested that up-regulation of SIRT1 induced by 17β-E2 inhibited apoptosis in hFOB1.19 osteoblasts. Yao et al. also reported that moderate overexpression of SIRT1 might suppress osteoblasts apoptosis through FOXO1/β-catenin pathway [25]. Forkhead box O (FOXO) transcriptional factor plays a significant role in bone metabolism by anti-apoptosis and anti-oxidative stress [62, 63]. FOXO3a, an important member of the FOXO family, is of special interest as it is the dominant subtype expressed in bone tissue [64]. SIRT1 deacetylates various transcription factors, including FOXO3a, thus playing significant roles in anti-apoptosis, metabolism and cell differentiation [35, 36, 64]. The present study suggested that up-regulation of SIRT1 increased FOXO3a and Bcl-2 expression, but decreased caspase-3 expression. Therefore, it is speculated that the up-regulation of SIRT1 induced by 17β-E2 might inhibit apoptosis in osteoblasts via FOXO3a activation. In this study, we demonstrated that the 17β-E2 administration could induce up-regulation of SIRT1 expression. Moreover, these findings suggested that the up-regulation of SIRT1 induced by 17β-E2 has the potential to protect against osteoporosis, and that the beneficial effect of SIRT1 depend, at least in part, on promoting osteoblasts autophagy via AMPK-mTOR pathway and inhibiting cell apoptosis via FOXO3a activation. Meanwhile, our findings presented a new visual angle and idea on the mechanism of SIRT1 in osteoporosis, and 17β-E2 combined with SIRT1 might represent a potential therapeutic target for osteoporosis. However, there are some limitations. Firstly, only one cell line hFOB1.19 was utilized *in vitro*. Secondly, we did not completely elucidate how SIRT1 regulates AMPK-mTOR and FOXO3a and the interactions between autophagy and apoptosis, therefore, further studies are needed.

## MATERIALS AND METHODS

### Cell culture and treatments

hFOB1.19 osteoblasts are bought from ATCC (Manassas, VA, USA). The cells were incubated with 10 % FBS in α-MEM/F12 medium (Invitrogen, MA, USA) at 37° C in 5 % CO_2_ and humidified atmosphere. 17β-E2 (Sigma, MO, USA) was prepared with different concentrations. Under normal cell growth conditions, they were subjected to 17β-E2 (0, 10^-8^, 10^-7^, 10^-6^ M) for a prescribed time period (0, 2, 4, 24, and 32 h). In combined administration, cells were precultured with the corresponding agonists or specific inhibitors for 2 h, and then treated with the optimal dosage (10^-6^ M) of 17β-E2 for 24 h. SIRT1 selective activator (SRT1720) was used at 10 μM [65, 66], SIRT1-specific inhibitor (EX527) at 10 μM [67, 68], autophagy inhibitor (3-MA) at 10 mM [69].

### Monodansylcadaverine (MDC) staining

MDC is called autophagy vacuoles specific probe, which is used to label autophagy-lysosomes [70–72]. The formation of autophagy vesicles is a hallmark of autophagy, marked by MDC and shown as bright color dots under a fluorescence microscopy [73]. MDC staining test was used for autophagy acidic vacuoles. The osteoblasts in the logarithmic growth phase were used, adjusted the cell density to inoculate l×l0^5^ cells/mL in the climbing slides in 24-well plates, cultured for 24 h. Osteoblasts were cultured with or without autophagy inhibitor 3-MA (10 mM) for 2 h followed by 17β-E2 (10^-8^ or 10^-6^ M) for 24 h [74]. The acidic vacuoles were rinsed with PBS three times, soaked in 4% PFA solution for 20 min, 0.05 mM MDC solution was added to each well, then cultured at 37° C for 1 h. Removed the MDC solution and rinsed with PBS 3 times. Then the slides were taken out and 3 visual fields were randomly selected under the fluorescence microscope (Olympus, Japan) to observe and take pictures, followed by analyzing the average relative fluorescence intensity with Image-Proclus 6.0 software.

### Immunofluorescence analysis

Immunofluorescence analysis of LC3 was performed as described previously [75]. Appropriate number of cells was cultivated in a confocal culture dish for 24 h. Cells were rinsed with PBS for three times. The osteoblasts were then soaked in 4% PFA for thirty min. After rinsing with PBS, cells were blocked at the non-lipid antibody site with 5% BSA in TBST. The membranes were cultured overnight at 4° C with specific rabbit anti-human LC3 (1: 200) primary antibodies. Then they were incubated with goat anti-rabbit IgG-HRP (1:3000, Sigma Inc., MO, USA) secondary antibody. After rinsing three times, they were observed under fluorescence microscope and the confocal laser scanning microscope (Olympus Optical Co., Ltd., Japan). Then the number of LC3 puncta was measured using Image J Software 1.48 (ML, USA).

### Transmission electron microscopy (TEM) observation

To detect autophagosomes, TEM remains the best and most powerful approach [76]. TEM analysis was conducted as described previously [77]. The osteoblasts were harvested and fixed in 2.5% glutaraldehyde in PBS buffer, then fixed in 1% osmium tetroxide in pure water for one hour. After gradient ethanol dehydration, the cells were embedded and sectioned. The samples were double-stained with uranyl acetate and lead citrate. Then, the autophagosomes of osteoblasts were observed under a Transmission Electron Microscopy (JEOL Ltd., Japan). The number of autophagosomes was determined using Image J Software 1.48 (ML, USA).

### MTT assay

Osteoblasts were cultured in 96-well plates at a cell density of 10^4^ cells per well. After attachment, cells were precultured with or without SRT1720 (10 μM) and EX527 (10 μM) for 120 min [65–68]. Then osteoblasts were incubated with 0 M or 10^-6^ M 17 β-E2 for 24 hours, 10 μL MTT reagent (5mg/mL) at 37° C for 3 h, and then cultured with dimethyl sulfoxide (DMSO) instead of culture medium for 4 h. Optical density was recorded using a microplate reader (Reagan Ltd., USA) at 490 nm.

### Cell apoptosis analysis

Osteoblasts apoptosis was determined by an Annexin V-FITC/PI apoptosis detection kit, and it was deployed according to the manufacturer’s protocols [78]. In short, cells (5 × 10^5^ cells/ sample) were collected, washed two times with PBS, and suspended in 500 μl binding buffer. Then samples were cultured with 5 μL of Annexin V-FITC and 5 μL PI solution at ambient temperature for fifteen min, and then immediately subjected to flow cytometry analysis using a flow cytometer (FACSCanto II, BD Biosciences, USA).

### RNA extraction, cDNA synthesis and real-time PCR assay

The mRNA level of SIRT1, LC3, Beclin-1, caspase-3, Bcl-2, FOXO3a in hFOB1.19 osteoblasts were detected by real-time PCR. Previous studies have shown that GAPDH mRNA expression is not regulated by 17β-E2 treatment [79, 80]. According to manufacturer’s instructions, total RNA was extracted from hFOB1.19 osteoblasts using TRIzol reagent (Invitrogen, USA). The RevertAid First-Strand cDNA Synthesis Kit (Thermo Scientific™, USA) was applied to reverse-transcribe RNA into cDNA. Then, using cDNA as template, the appropriate specific primers and real-time PCR kit (Takara Inc., China) were used to detect the mRNA level. The specific primer sequences of SIRT1, caspase-3, LC3, FOXO3a, Bcl-2 and Beclin-1 were listed in Table 1. GAPDH was used for normalization and served as the internal reference. The relative mRNA expression levels were quantitated using the 2^-ΔΔCt^ method.

**Table 1:** Primer and probe sequences of all genes used in RT-PCR.

**Primers**	**Forward primer (5′–3′)**	**Reverse primer (5′–3′)**
SIRT1	GTTGTGTGCCTTCGTTTTGGA	AGGCCGGTTTGGCTTATACA
LC3	CTCTCTGAGCCTTAGGTGCC	ACTCGTGGGGTGACCATTTC
Beclin-1	GAATGGAGGGGTCTAAGGCG	CCTCTTCCTCCTGGCTCTCT
caspase-3	GATGTGGACGCAGCCAACCTCA	TCCGGCAGTCGCCTCTGAA
FOXO3a	CGTTGTTGGTTTGAATGTGGG	GGTTTTCTCTGTAGGTCTTCCG
GAPDH	AGTCTACTGGCGTCTTCACC	CCACGATGCCAAAGTTGTCA

### Protein preparation and Western blot analysis

After administration, hFOB1.19 osteoblasts were lysed with NP-40 lysis buffer and the extracts were centrifuged to collect the supernatant containing total protein. Then a Pierce BCA Protein Assay Kit (Thermo Scientific™, USA) was applied to measure the protein concentration, according to the manufacturer’s instructions [81]. The protein samples were separated by SDS-PAGE gels (10% SDS)for 100 min at 110 V and transferred to a PVDF membrane [82]. Then, the membranes were blocked for one hours using 5% skim milk/ BSA at ambient temperature and incubated with primary antibodies (SIRT1, AMPK, p-AMPK, LC3, Bcl-2, caspase-3, Beclin-1, p-mTOR (Ser2448) and β-actin antibodies) at 4° C overnight. Phosphorylation of the m-TOR Ser2448 site is a direct target of the anti-p-mTOR antibody. Then, the membranes were incubated with corresponding secondary antibodies for 60 min at ambient temperature. The BeyoECL plus kit (Beyotime Bio Ltd., China) was used to visualize the proteins. β-actin was used as an internal reference. The bands densitometric analysis was measured by Image J Software 1.48 (ML, USA) [83].

### Statistical analysis

Results were represented as means ± standard deviation (S.D.). The experiments were repeated three times independently. Statistical significance was analyzed by Student’s t-test, one-way Analysis of Variance (ANOVA), followed by Student-Newman-Keuls test. *P* < 0.05 was represents statistically significant difference. Statistical analysis was performed with the assistance of SPSS 15.0 Software (SPSS, Inc., USA) for Microsoft Windows.

## Supplementary Material

Supplementary Figure 1
