# Supplementary material for: Up-regulation of SIRT1 induced by 17beta-estradiol promotes autophagy and inhibits apoptosis in osteoblasts
Source: Aging (Albany NY). 2021 Oct 28;13(20):23652–71. doi: 10.18632/aging.203639 (PMC8580331; doi:10.18632/aging.203639)
Supplement: Supplementary Figure 1 [file aging-13-203639-s001.pdf]

SUPPLEMENTARY FIGURE

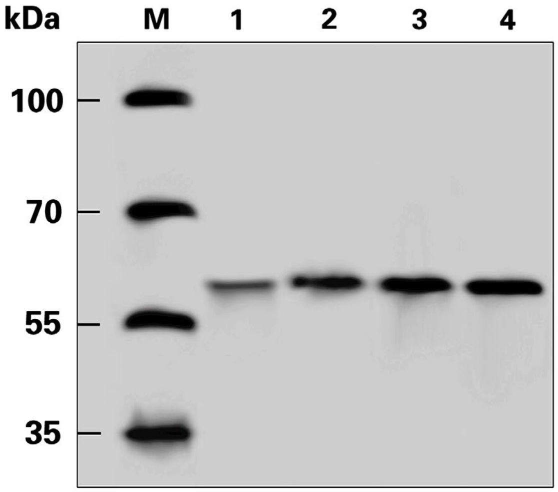

Supplementary Figure 1. The original gel acquisition of the Western blot shown in Figure 1A. M: marker protein, 1: control group (17β-E2 : 0 M), 2: 17β-E2 (10^{-8} M), 3: 17β-E2 (10-7 M), 4: 17β-E2 (10-6 M), SIRT1 = 60 kDa.
